# δ-Sarcoglycan-deficient muscular dystrophy: from discovery to therapeutic approaches

**DOI:** 10.1186/2044-5040-1-13

**Published:** 2011-03-17

**Authors:** Alison M Blain, Volker W Straub

**Affiliations:** 1 Institute of Genetic Medicine, Newcastle University, International Centre for Life, Central Parkway, Newcastle upon Tyne, NE1 3BZ, UK

## Abstract

Mutations in the δ-sarcoglycan gene cause limb-girdle muscular dystrophy 2F (LGMD2F), an autosomal recessive disease that causes progressive weakness and wasting of the proximal limb muscles and often has cardiac involvement. Here we review the clinical implications of LGMD2F and discuss the current understanding of the putative mechanisms underlying its pathogenesis. Preclinical research has benefited enormously from various animal models of δ-sarcoglycan deficiency, which have helped researchers to explore therapeutic approaches for both muscular dystrophy and cardiomyopathy.

## δ-Sarcoglycanopathy

### Historical and clinical aspects

In 1954, Walton and Nattrass described a group of patients who shared a similar pattern of proximal muscle weakness and whose symptoms could not be assigned to any of the known muscular dystrophies (MDs) of that time [1]. This highly heterogeneous group of patients was broadly characterised as having the limb-girdle muscular dystrophies (LGMDs). It was not until the discovery of dystrophin as the relevant protein missing in Duchenne muscular dystrophy (DMD) [2,3] and the isolation of the dystrophin glycoprotein complex (DGC) [4] that this group was further stratified according to the molecular defect. It is now known that a subgroup of patients who present with an LGMD phenotype harbour mutations in genes encoding four of a family of six transmembrane proteins called the sarcoglycans (SGs).

LGMD2F was the fourth of the sarcoglycanopathies (LGMD2C to LGMD2F) to be characterised when the causative mutation in the δ-SG gene was identified in a group of Brazilian LGMD patients with a DMD-like presentation whose disease was linked to a region on chromosome 5 (q33-34) [5-7]. Since then, a large number of mutations causing both LGMD2F and δ-SG-associated cardiomyopathy have been described (Leiden Muscular Dystrophy database: http://www.dmd.nl/).

The age of onset in LGMD2F can vary from early childhood to adulthood. Most patients present with progressive weakness and wasting of the proximal muscles and elevated serum creatine kinase in the first decade of life [8]. General survival depends on cardiac and respiratory involvement and patients can die, sometimes even at early stages of the disease, because of severe dilated cardiomyopathy or chest infections and respiratory failure [9,10]. The diagnosis is based initially on examination of a muscle biopsy (which exhibits dystrophic features with reduced SG expression) and is confirmed by genetics [11]. Sarcoglycanopathy has, as a whole, a relatively low prevalence in nonconsanguineous populations (2.27/100,000 is a recent estimate) [12,13], and whilst δ-sarcoglycanopathy seems to be the rarest of this group of MDs [14], the understanding of its pathogenesis has implications for the development of therapeutic approaches for both patients with MD and patients with cardiomyopathy.

While patients with LGMD2F most commonly present with skeletal muscle weakness and only develop cardiomyopathy at later stages of their disease, δ-SG mutations have also been found in patients with primary hereditary dilated cardiomyopathy with no significant skeletal muscle symptoms [15-18]. It has been suggested that at least one of these mutations could cause mild pathology in heterozygous carriers [18] or could even show a dominant mode of inheritance [15], but further evidence is necessary to clearly show that both recessive and dominant mutations are responsible for δ-SG-deficient cardiomyopathy [19].

### δ-Sarcoglycan

The δ-SG gene contains nine exons spanning a 433-kb region of genomic DNA. It encodes a 35-kDa, single-pass, type II transmembrane glycoprotein. To understand its role in the pathology of MD and cardiomyopathy, it is important to consider it as one of a family of six SG proteins, four of which, α-, β-, γ- and δ-SG, are associated with forms of autosomal recessive LGMD and cardiomyopathy, and one of which, ε-SG, is associated with a form of myoclonus dystonia, whereas no disease has been associated with ζ-SG to date. γ- and δ-SG share a similar structure with a conserved sequence in the C-terminal domain, which is essential for their localisation to the plasma membrane, and an N-terminus that is important for SG-SG interaction [20]. It is generally accepted that, in skeletal muscle, at least α-, β-, and γ-SG form a tetrameric complex with δ-SG. There is also evidence that ε-SG can replace α-SG in striated muscle to form a second SG complex [21].

Coimmunoprecipitation studies have shown a strong interaction between δ- and β-SG [22], which is thought to form a central core that is necessary for the delivery and retention of other SGs to the cell membrane [23,24]. This hypothesis is supported by the fact that patients with mutations in either δ- or β-SG appear to have a complete loss or a strong reduction of the whole SG complex on examination of muscle biopsy samples [14,25,26]. It is thought that in smooth muscle, ε-SG functionally replaces α-SG to form a unique complex analogous to that in skeletal muscle [27,28]. The more recent discovery of ζ-SG in skeletal and smooth muscle [29] has led to the prediction of other models of SG complex conformation [30-32].

The various forms of SG complexes all seem to form an integral part of the dystrophin-glycoprotein complex (DGC) [33], the function of which has best been characterised in skeletal muscle, where it forms a mechanosignalling link between the F-actin cytoskeleton and the extracellular matrix. The DGC confers structural stability to the sarcolemma, and dissociation of the complex by loss of one of its components renders the muscle more susceptible to contraction-induced damage. Dissociation of the DGC complex is also thought to have widespread implications for a number of signalling processes, as it is closely associated with a diverse range of molecules, such as neuronal nitric oxide synthase (nNOS) [34-36] and members of the integrin family of signalling proteins [37-39].

An alternative splice variant of δ-SG, SG3, has been localised to the membrane of the sarcoplasmic reticulum (SR), where it forms a complex homologous to that at the sarcolemma [40]. It is hypothesised that this SR complex plays a role in regulating calcium movement across the SR membrane, which is important for the maintenance of calcium homeostasis.

Although the SGs are expressed primarily in muscle, there is evidence that they are also expressed in other tissues, such as myelin [41], adipocytes [42], kidney and lung epithelial cells [28] and the retina [43]. The functional relevance of SG expression in these tissues is currently unclear, however, as patients with LGMD2F do not appear to show neuropathic, central nervous system or cognitive impairments, nor do they have any overt aberrations in glucose metabolism or kidney and lung function.

## Animal models of δ-SG deficiency

There are a number of animal models of δ-sarcoglycanopathy that have aided in the understanding of its pathogenesis (Table 1). The cardiomyopathic hamster is the oldest and most well-characterised of these.

**Table 1:** Animal models of δ-sarcoglycan deficiency^a^

Species	Genesis	Effect on protein/DGC components	Phenotype
Hamster			
BIO 14.6	Naturally occurring, autosomal recessive mutation (30-kb deletion in exons 1 and 2) [44]	Loss of δ- and β-SG Reduction of α- and γ-SG [47,54] Reduction in α-dystroglycan [47] Normal dystrophin	Compensatory hypertrophic CM leading to dilated CM Sarcolemmal damage (increased EBD uptake)
TO-2	Cross-breeding (30-kb deletion in exons 1 and 2)	Complete loss of SG complex Translocation of dystrophin to cytoplasm [150]	Severe dilated CM LV dysfunction from 8 weeks [151] Gait disturbances [152]
J2N-k	Cross-breeding (BIO 14.6 × golden hamster, then consecutive sib mating) [153]	Uncharacterized?	Cardiac contractile dysfunction Dilated CM from 20 weeks [90] Elevated CK level
UMX7.1 or CHF147	Cross-breeding (BIO 14.6 × normal controls) [154]	Uncharacterized?	Dilated CM Progressive LV dysfunction [155] Reduced life expectancy (190 days) Early skeletal muscle pathology (10 to 15 days) Focal necrosis Unselective muscle involvement
Mouse			
*Sgcd*^-/- ^(C57BL6 background)	Transgenic (vector-mediated, knockdown-targeted replacement of exon 2, which encodes the entire TM domain and part of the intracellular domain) [57]	Loss of whole SG complex and sarcospan	Limb-girdle pattern of muscle involvement Focal areas of necrosis Cardiomyopathy from 8 weeks, ECG abnormalities Increased probability of spontaneous death at 6 months
*Sgcd*^-/- ^(129SvJ/129SvEms- +^Ter^/J background)	Transgenic (vector-mediated replacement of exon 2; homozygotes generated from heterozygote matings) [56], resultant δ-SG mRNA lacking 201-bp region.	Loss of all SGs (including ε-SG) in muscle microsomes on immunoblot despite normal levels of transcription	Premature death: only 50% survival at 28 weeks Elevated CK Regional degeneration/regeneration, calcification, inflammatory infiltration, perivascular fibrosis and increased EBD uptake on muscle histology Cardiac histological changes at 12 weeks Reduced force generation in response to eccentric contractions
*Drosophila*			
Line 840	Engineered (large deletion by P element excision) [65]	Loss of whole δ-SG protein Effect on other DGC components uncharacterized	Shortened lifespan Progressive impairment in locomotive ability Reduced heart tube function Abnormal flight muscles No regeneration
Line 28	Engineered (small deletion by P element excision) [65]	Loss of cytoplasmic region of δ-SG only Effect on other DGC components uncharacterized	Mild Near-normal lifespan Normal cardiac function Normal locomotive function
*Caenorhabditis elegans*			
F07H5.2	RNA interference (animals fed or injected with dsRNA corresponding to 500- to 700-bp exon-rich region) [64]	Uncharacterized?	Phenotype similar to dystrophin KO (*dys-1*) (bending of head with forward movement, hyperactivity, hypercontraction)
Zebrafish			
N/A	Morpholino knockdown of δ-SG [67]	Downregulation of δ-, β- and γ-SGs	Disorganized muscle development Reduced movement 5 dpf
N/A	Morpholino knockdown of δ-SG [66]	Uncharacterized?	Severe abnormality of skeletal and cardiac muscle Delayed cardiac development and abnormal cardiac differentiation Dead by 5 dpf

^a^CM, cardiomyopathy; dpf, days postfertilization; KO, knockout; SG, sarcoglycan; EBD, Evans blue dye; LV, left ventricular; CK, creatine kinase; DGC, dystrophin-glycoprotein complex; ECG, electrocardiogram; *Sgcd*^-/-^, δ-sarcoglycan-deficient; dsRNA, double-stranded RNA.

### The cardiomyopathic hamster: a model of sarcoglycanopathy *in cognito*

The BIO 14.6 hamster strain was established in 1962 [44] and was studied for over three decades as a model of cardiomyopathy, but it was only after the discovery of the causative mutation in LGMD2F patients that two independent laboratories demonstrated a large deletion in the 5' end of the δ-SG gene in these animals [45,46]. The hamster was therefore heralded as the first animal model of sarcoglycanopathy.

Like the majority of LGMD2F patients, the hamster lacks sarcolemmal δ-SG expression and has a concomitant reduction in the other components of the SG complex [47]. Cardiac damage could be seen in the heart from 5 weeks of age, indicating the early onset of pathology in cardiac muscle compared to skeletal muscle [47]. Assessment of cardiac haemodynamics in the hamster over its lifetime (240 days) indicates a cardiomyopathic phenotype with decreased stroke volume and cardiac output (CO) as well as increased ventricular mass [48].

At least three additional strains of hamsters, the TO-2, CHF147 (formally VMX7.1) and J2N-k strains, have since arisen from the original BIO 14.6 strain. The TO-2 strain is particularly interesting, as these hamsters exhibit a more severe cardiomyopathy and a shorter lifespan than their BIO 14.6 counterparts. In addition, while both strains share the same 30-kb deletion in the δ-SG gene, the BIO 14.6 strain develops a hypertrophic cardiomyopathy, whilst the TO-2 hamsters tend to develop a severe dilated cardiomyopathy with a predominantly necrotic mode of pathology [49,50]. This presents the intriguing possibility of the presence of a genetic modifier within the TO-2 hamster. Indeed, a missense mutation in the mitochondrial DNA of hypertrophic TO-2 hamsters, which is not present in the BIO 14.6 strain, has been identified [51]. Other mapping approaches have also led to the identification of genetic modifiers of fibrosis, membrane leak and muscle-specific modifying loci in another mouse model of sarcoglycanopathy [52,53]. Genetic modifiers may therefore partly account for heterogeneity in both clinical presentation and muscle involvement in patients with LGMD2F.

Another interesting difference between these two strains of hamsters is the expression pattern of the SGs. Whilst the cardiac muscle of BIO 14.6 stains weakly for α- and γ-SGs, the TO-2 strain does not stain for any of the SGC components [54]. The TO-2 strain therefore provides a 'cleaner' background for the assessment of transduction efficacy in gene transfer studies. Again, genetic modifiers may account for this discrepancy between strains of δ-SG-deficient hamsters and also in the rare patients who display a milder phenotype with partial retention of the SG complex at the sarcolemma [55].

### The δ-sarcoglycan-null mouse

While the hamster remains a useful, naturally occurring model for δ-SG-deficient cardiomyopathy, δ-SG-deficient (*Sgcd*^-/-^) mice have also been generated [56,57]. From a young age, these mice develop typical histological features of MD in most of their skeletal muscles. As in the hamster, the diaphragm shows a particularly severe pathology with abnormalities evident from as early as 4 weeks of age [57].

*Sgcd*^-/- ^mice also develop cardiomyopathy from around 8 weeks of age, with focal areas of fibrosis that were thought to be related to abnormalities in the coronary vasculature [57]. The primacy of these vascular events in the development of MD is contentious, however. Functionally, 8-week-old *Sgcd*^-/- ^mouse hearts show electrocardiographic abnormalities [57], and by 16 weeks, they develop a well-compensated cardiomyopathy, with reduced contractility but preserved ejection fraction (EF) and CO [58,59]. However, by 32 weeks of age, there is a deterioration in pathology such that the left (and right) EF is significantly reduced, with evidence of right ventricular dilation, which is indicative of pulmonary dysfunction [60]. Pulmonary dysfunction is consistent with the severe pathology observed in the diaphragms of *Sgcd*^-/- ^mice and is also a feature of advanced stage LGMD [9,61]. The *Sgcd*^-/- ^mouse, therefore, like the hamster, recapitulates many of the important features of the human disease, making it a useful preclinical research tool.

### Fly, worm and fish models of δ-SG deficiency

**Figure 1:**
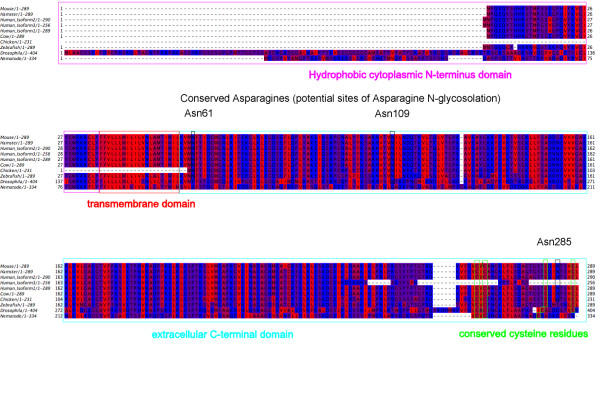
**Alignment of δ-sarcoglycan (δ-SG) in different species illustrating conserved regions**. δ-SG is a transmembrane protein (residues are coloured according to hydrophobicity, with the most hydrophobic residues shown in red and the least hydrophobic residues shown in blue) with several highly conserved regions. Human δ-SG is differentially spliced to produce three proteins, which differ at their C-terminus.

Despite their early evolutionary divergence from mammals, δ-SG deficiency orthologues have been identified in *Drosophila*, zebrafish and the nematode *Caenorhabditis elegans *[62-64] (Figure 1). These organisms are easy to manipulate genetically while being cheap to breed and maintain. They have been particularly useful in indicating the protein domains that are most important to δ-SG function [65] and for investigation of the developmental expression of δ-SG in different tissues [63,66,67]. RNA interference work in *C. elegans *has also generated important data suggesting the involvement of calcium channel disruption in the pathology of MD [68].

## Putative disease mechanisms

### Calcium overload: a unifying mechanism?

**Figure 2:**
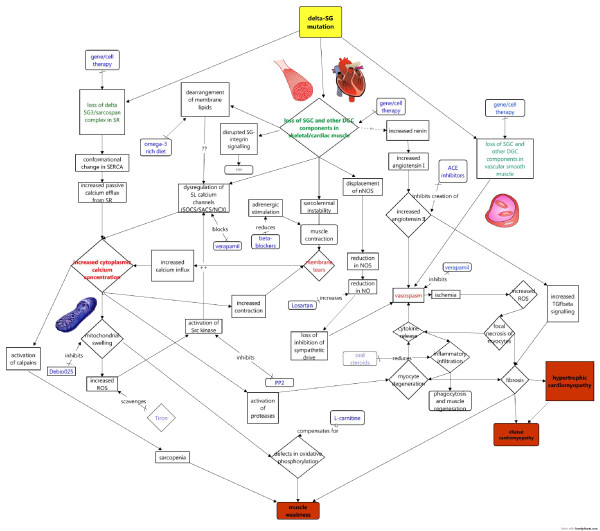
**Putative pathomechanisms involved in δ-sarcoglycanopathy revealed by studies of the animal models of the disease**. Disease mechanisms suggested by animal studies, and their possible relationships to each other, are presented schematically. Pivotal events in the development of pathology are highlighted in red, and potential therapies are shown in blue. Faded boxes represent therapies that have had some success in animal models of other muscular dystrophies or are currently used in Duchenne muscular dystrophy and as such may be potentially useful in the treatment of δ-sarcoglycanopathy.

Studies in animal models of δ-sarcoglycanopathy have revealed a baffling array of pathological changes, which have been targeted by various drug therapies (Figure 2). However, many of these mechanisms can be traced to an upstream elevation in intracellular calcium. Indeed, calcium overload is sufficient to elicit a MD phenotype in mice [69]. In δ-sarcoglycanopathy, calcium overload is thought to occur through two main mechanisms: through membrane tears due to loss of sarcolemmal stability and through abnormalities in calcium channels of the sarcolemmal or sarcoplasmic reticulum number or function.

#### Loss of membrane integrity

The membrane instability theory of muscle pathology predicts that the loss of components of the DGC leads to susceptibility to contraction-induced damage, increased influx of calcium and ultimately cell degeneration. There is evidence to suggest that this is a common primary pathomechanism in a number of MDs [70]. More specifically, membrane fragility and abnormalities ('Δ lesions') have been noted in patients and in animal models of sarcoglycanopathy [71,72]. A study using the TO-2 hamster suggested that this sarcolemmal fragility is due to a secondary loss of dystrophin [73]. Dystrophin loss is not a feature of all strains of δ-SG hamster [47], however, suggesting that loss of SGC alone is sufficient to produce sarcolemmal instability. Sarcolemmal damage can be easily assayed in animal models by means of Evans blue dye (EBD), which cannot enter intact cells. Mouse and hamster models of δ-sarcoglycanopathy both show evidence of increased EBD uptake, in both heart and skeletal muscles [47,57,58].

#### Calcium channel dysregulation

Besides the structural role of δ-SG, it has been proposed that loss of the components of the DGC can produce a more specific alteration in calcium influx via disruption of calcium channels. There have been no studies conducted to date suggesting a direct interaction between the SG complex and calcium channels; however, electrophysiological studies of myocytes isolated from dystrophic hamsters have shown the presence of abnormal calcium currents and increased intracellular calcium levels even in the absence of mechanical stress [74]. Attempts to identify the origin of these abnormal currents in cardiomyocytes from δ-SG-deficient hamsters have yielded contradictory evidence. While it has been suggested that L-type calcium channels (LCCs), the predominant calcium channel in the heart, are neither more active nor overexpressed in the hamster [75-77], LCC antagonists (tranilast, diltiazem and FK506) have had a protective effect on muscle [76]. Also, increased enrichment of the pore-forming subunit of the LCC has been found in microsomes of myopathic hearts, although not from ventricular myocytes [78].

Other electrophysiological studies have identified T-type calcium channels as being important in the abnormal calcium homeostasis of δ-SG-deficient hamster cardiomyocytes [79,80]. Indeed, T-type channel blockers that have low LCC activity (efonidipine and mibefradil) improve cardiac function in the hamster [81,82]. However, a comparison of mibefradil and verapamil efficacy in preventing the dystrophic process in hamsters showed no benefit of mibefradil [83].

In skeletal muscle, the calcium entry required for contractile activity enters via store-operated and stretch-activated calcium channels (SOCs and SACs, respectively) [84]. Elevated SAC currents have been detected in δ-SG-deficient myotubes [85], and although transient receptor potential canonical 1 (TRPC1) has been suggested as a candidate SAC involved in the pathogenesis of the *mdx *mouse model of DMD and cardiomyopathy [86,87], there have been no studies of this channel in δ-sarcoglycanopathy.

Some evidence points towards there being abnormalities in internal calcium homeostasis across the SR membrane in δ-SG-deficient animals [40,88]. Indeed, a recent study of *Sgcd*^-/- ^mice suggested that two isoforms of δ-SG (SG1 and SG3) play a role in the stabilisation of γ-SG and sarcospan expression in the T-tubule and SR membrane [89]. The authors showed that *Sgcd*^-/- ^mice possess a conformational change in SERCA1 Ca^2+^-ATPase (sarco(endo)plasmic reticulum ATPase 1), which alters resting calcium homeostasis. Additionally, it has been demonstrated that SG3 is located in close proximity to voltage-sensing dihydropyridine receptors that play a role in regulating calcium release via ryanodine receptors in the SR [40]. These findings may explain earlier observations in the J2N-k hamster which suggested that SR abnormalities contribute to contractile dysfunction in these animals [90]. Therapeutic strategies for δ-sarcoglycanopathy, then, should perhaps aim to correct internal calcium homeostasis as well as reduce calcium influx across the sarcolemma.

### Downstream consequences of calcium overload

#### Oxidative Stress and mitochondrial dysfunction

Mitochondrial swelling is commonly observed in MD, and early electromicroscopy studies in tissues and cells isolated from the cardiomyopathic hamster revealed several abnormalities in the fine structure of the mitochondria [91,92]. Other studies have suggested, however, that these changes are secondary to increased intracellular calcium and that mitochondrial swelling due to calcium overload can lead to myocyte necrosis [93-96]. Although latent mitochondrial dysfunction can be observed at high heart rates in younger δ-SG-deficient hamsters, it is ameliorated by verapamil, lending further support to calcium involvement [97]. Increased reactive oxygen species (ROS) production due to mitochondrial overload is also thought to cause activation of Src kinase and subsequent activation of TRPC1 at the sarcolemma [86]. Mitochondrial dysfunction may therefore feed back to calcium channels on the sarcolemma to exacerbate already elevated intracellular calcium levels. ROS scavengers such as tiron, which have already been demonstrated to have some success in *mdx *mice [86], may therefore be useful in halting this vicious cycle in δ-sarcoglycanopathy.

Recently, it has been demonstrated that overactivation of the mitochondrial transition pore (MPTP) in δ-SG-deficient hamster cardiomyocytes renders the mitochondria of these hamsters leaky to calcium influx and susceptible to mitochondrial swelling [93]. Mitochondrial swelling has been targeted directly as a strategy for the treatment of patients with LGMD2F. Deletion of the gene encoding cyclophilin D, a component of MPTP, renders mice unsusceptible to mitochondrial swelling and ameliorates pathology in *Sgcd*^-/- ^mice [98]. Indeed, treatment with a cyclophilin D inhibitor (Debio-025) is effective in reducing pathology in *Sgcd*^-/- ^mice and also in the *mdx *mouse model of DMD [98]. This may suggest that mitochondrial swelling is a common downstream cause of pathology in both LGMD2F and DMD.

In addition to calcium overload, defects in carnitine transport have been implicated in mitochondrial dysfunction in δ-sarcoglycanopathy. Reduced carnitine levels can be detected at a prepathological time point in δ-SG-deficient hamsters [99]. L-Carnitine facilitates the transport of fatty acids into mitochondria for energy production via oxidation, and carnitine deficiency syndrome results in muscle weakness, so it can be envisaged that deficiency in this molecule could account for some of the symptoms of LGMD2F. There have been a number of studies that have suggested that supplementation with L-carnitine or propionyl-L-carnitine can have positive effects on δ-SG mouse hearts and skeletal muscle, in terms of both energy metabolism and pathology [100-106]. Similarly, other dietary supplements such as selenium, taurine and coenzyme Q_10 _[106,107], as well as mild exercise regimes [108-110], have shown a beneficial effect on mitochondrial function and hence muscle pathology in the cardiomyopathic hamster, providing further evidence of lifestyle factors which may influence disease progression. Therapeutically, mitochondrial dysfunction may therefore represent a rather downstream but relatively easily targeted disease mechanism.

#### Vascular dysfunction

Although δ-SG is expressed in vascular smooth muscle, vascular dysfunction as a primary cause of MD pathology is controversial. Adenoviral transfer of δ-SG into *Sgcd*^-/- ^mice was sufficient to prevent MD, although vascular smooth muscle was not transduced, suggesting that vascular dysfunction is not a primary cause of pathology [111-113]. Early data which showed that the vasodilator verapamil could ameliorate 'microinfarcts' and focal areas of fibrosis in the *Sgcd*^-/- ^mouse and the BIO 14.6 Syrian hamster [112-115] was originally taken as evidence in support of the vasospasm theory. However, the interpretation of these data is muddied by the fact that verapamil can affect LCCs in cardiomyocytes as well as in smooth muscle and hence may normalise a myocyte-intrinsic defect [116]. In support of this hypothesis, a γ-SG-deficient mouse with no perturbation in vascular smooth muscle SGs develops cardiomyopathy and vasospasms, presumably secondary to cardiomyocyte degeneration [117]. Furthermore, Shimizu *et al*. [118] showed that progressive myocyte loss is responsible for deterioration of cardiac function in BIO TO-2 hamsters, while impaired vascular regeneration may be responsible for progressive remodelling. The general view is therefore that vascular spasm is an important mechanism that contributes to the progression of LGMD2F, but that it should be seen as a secondary consequence of myocyte injury through calcium-activated processes such as increased proteolysis by calpains [119] and mitochondrial dysfunction.

One of the factors that may make δ-SG-deficient animals particularly susceptible to vasospasm and focal ischaemia is disruption of nNOS. Displacement of nNOS from the sarcolemmal membrane has been noted in patients with DMD and more recently in LGMD patients with SG mutations and animal models of sarcoglycanopathy [35,120]. nNOS is associated with dystrophin at the sarcolemmal membrane [34] and generates NO, a molecule with a pivotal role in modulating blood flow [121], through a calcium-dependent process. It is thought that disruption of nNOS renders the muscle more susceptible to focal ischaemia and damage by superoxides [121,122]. That nNOS has a role in vascular dysfunction in δ-sarcoglycanopathy is further evidenced by the observation that drugs which increase myocardial NO (for example, simvastatin, losartan) also improve cardiomyopathy in the hamster [123-125].

Overactivation of the renin-angiotensin system may also contribute to vasospasm in the δ-SG-deficient hamster [123,126-128]. Indeed, the use of angiotensin-converting enzyme inhibitors (ACEi) and aldosterone antagonists has demonstrated some success in ameliorating cardiac and diaphragmatic pathology and function in both hamster and mouse models of δ-SG deficiency [129-132]. ACEi have well-established vasodilative actions and form part of the cardiac management for LGMD2F patients. They may have additional benefit, however, because of their less well-characterised antifibrotic actions [133].

## Therapeutic strategies

### Current therapeutic strategies

There is currently no cure for LGMD2F and other muscular dystrophies involving the DGC. Current clinical management is similar to that described in published care guidelines for DMD [11,134] and concentrates on management of cardiac and respiratory symptoms. Steroids are not routinely used in the treatment of LGMD2F, however, despite anecdotal reports of their efficacy in sarcoglycanopathies.

### Replacing defective genes or proteins

#### Viral vector-mediated gene transfer

Intuitively, correction of the genetic defect or replacement or substitution of δ-SG protein is the simplest approach to tackling LGMD2F. Indeed, δ-SG has a much shorter coding sequence than dystrophin and as such may not lead to many of the difficulties that are encountered in gene therapy for DMD. Studies in *mdx *mice, however, have shown that cardiac tissue is particularly difficult to target and skeletal muscle-centric treatment carries the danger of producing a more aggressive cardiac phenotype [135].

Intramuscularly administered adeno-associated viral (AAV) vectors have successfully restored cardiac and skeletal muscle SG membrane proteins in the δ-SG-deficient mouse and hamster, slowing functional deterioration [136-140]. Furthermore, it has been demonstrated that AAV transduction can be efficient and long-lived [141]. From a translational point of view, therapies that can be delivered systemically are more feasible, and it has been shown that systemically delivered AAV vectors are also efficient in reconstituting the SGC while reducing functional deterioration in the cardiac muscle of δ-SG-null mice and, encouragingly, in relatively old BIO 14.6 hamsters [142,143]. Work using virus display peptide libraries has suggested that systemically administered AAV vectors can also be specially selected to 'home' in to specific tissues, allowing greater control of where the vector is delivered [144].

Despite these encouraging results from various animal studies, the therapeutic approach is not easily translatable to humans because of potential immunogenic and toxic complications; however, there have already been promising results in patients with α-SG deficiency [145,146].

#### Autologous and nonautologous cell-based therapies for δ-SG deficiency

Skeletal muscle is a source of myosphere-derived progenitor cells (MDPCs), which are multipotent cells that express a number of embryonic stem cell markers and differentiate into vascular smooth muscle cells and mesenchymal progeny. They have been shown to enhance neoangiogensis and restore δ-SG expression in the vasculature of *Sgcd*^-/- ^mice [147]. MDPCs also promote secretion of paracrine effectors such as hepatocyte growth factor and stromal cell-derived factor 1, which are beneficial to cardiac function. Stem cells from patients can be pathologically impaired, however, such that other, nonautologous cell therapy approaches may be necessary.

Nonautologous cell therapy circumvents some problems associated with the quality and quantity of cells isolated for an autologous cell therapy approach. However, human leukocyte antigen incompatibility of nonautologous stem cells normally necessitates the use of immunosuppressive drugs, which have unattractive side effects. A recent study in TO-2 hamsters suggested that mesenchymal stem cells, which express a nonimmunogenic phenotype, may have potential as a source for nonautologous cell therapy that does not require immunosuppression [148]. Recently, intramyocardial injection with human umbilical cord mononuclear cells was shown to decrease fibrosis and increase short-term cardiac function in TO-2 hamsters [149]. Again, as with AAV gene transfer, approaches that have had success in animals may not be acceptable to the human immune system.

## Conclusions

It is evident that δ-SG deficiency causes a complex and multifactorial pathology for which a combinatorial therapeutic strategy may be necessary. The clinical manifestation of the disease is heterogeneous, with evidence of genetic modifiers. There are currently a number of potential therapeutic agents which target downstream defective pathways, but correction of the primary defect via gene or cell therapy may prove to be the most effective treatment strategy in the future. A focus on translational research is most important to ensure that patients benefit from our improved understanding of the pathophysiology implicated in δ-SG deficiency.

## Competing interests

The authors declare that they have no competing interests.

## Authors' contributions

AMB and VWS participated in the research of the literature for this review and in the preparation of the manuscript and figures. Both authors read and approved the final manuscript.
